# Phase 1 study of the safety, tolerability, and pharmacokinetics of a synthetic macrocyclic peptide antibiotic (BRII-693) in healthy adult participants

**DOI:** 10.1128/aac.01288-24

**Published:** 2024-12-09

**Authors:** Michael Watkins, Yali Zhu, David C. Griffith, Jeffery S. Loutit, David Margolis, Peidi Gu

**Affiliations:** 1 Brii Biosciences Inc., Durham, North Carolina, USA; 2 Qpex Biopharma Inc. 656306 , San Diego, California, USA; Providence Portland Medical Center, Portland, Oregon, USA

**Keywords:** antibiotic, phase 1, pharmacokinetics, safety

## Abstract

BRII-693 is a next-generation intravenous (IV)-administered synthetic macrocyclic peptide antibiotic for infections caused by drug-resistant gram-negative pathogens. This single-center, randomized, double-blind, placebo-controlled phase 1 study investigated the safety, tolerability, and pharmacokinetics (PK) of single and multiple ascending doses of BRII-693 in 104 healthy participants. In single-dose cohorts, 10–400 mg of BRII-693 was evaluated in eight participants (six active; two placebo) per cohort. In the 7-day repeat-dose cohorts, 100–200 mg of BRII-693 was evaluated in eight participants (six active; two placebo) per cohort. In two 14-day repeat-dose cohorts, 150 mg of BRII-693 was evaluated in 12 participants (10 active, two placebo) each of non-Chinese and Chinese descent. No participant reported a severe or serious adverse event (AE) or an AE leading to death. Across all cohorts and for non-Chinese and Chinese participants, most AEs were mild. C_max_ and area under the concentration-time curve (AUC) increased in a dose-proportional manner over the dose range of single- and repeat-dosing. Mean *t*_1/2_ was 2.58–4.37 hours and generally similar across single doses. An accumulation of exposure was observed following multiple doses with an accumulation ratio of 1.5 to 1.7 which was within the expected 1.3 to 2.5 range at steady state. Mean total clearance (CL) was similar between single and multiple dose administration, suggesting time-independent pharmacokinetics (PK). PK exposure was statistically equivalent between non-Chinese and Chinese participants. This phase 1 study demonstrates a favorable safety, tolerability, and PK profile of BRII-693 in healthy non-Chinese and Chinese participants.

**CLINICAL TRIALS:**

This study is registered with ClinicalTrials.gov as NCT04808414.

## INTRODUCTION

The 2024 update of the World Health Organization Bacterial Priority Pathogens List has ranked carbapenem-resistant Enterobacterales (CRE) and carbapenem-resistant *Acinetobacter baumannii* (CRAB) as critical priority and carbapenem-resistant *Pseudomonas aeruginosa* as high priority. Some progress has been made in the successful development of new agents to address drug-resistant infections, particularly intravenous (IV) agents for resistant gram-negative bacteria. While some of these agents have addressed urgent threats like CRE, their activity is largely confined to strains producing serine carbapenemases (1). Out of the recently approved agents, only cefiderocol has activity against metallo beta-lactamase-producing CRE as well as activity against *A. baumannii and P. aeruginosa* (2). Sulbactam-durlobactam has demonstrated its activity against carbapenem-resistant *A. baumannii–calcoaceticus* complex in a global phase III trial and was approved by FDA in 2023 (3) but does not have activity against serine or metallo beta-lactamase-producing CRE or *P. aeruginosa*. The evolving challenges of antibiotic resistance underscore the urgent need for broader spectrum antibiotics against carbapenem-resistant pathogens.

Polymyxin B and colistin were commonly used in clinical treatment until the 1980s when the associated nephrotoxicity caused a decline in usage (4–6). However, the increasing prevalence of multidrug-resistant gram-negative bacteria resulted in increased use of polymyxin B and colistin as salvage therapy (7) despite the known nephrotoxicity and neurotoxicity associated with polymyxins (8).

BRII-693 (formerly QPX9003) is an achievement in drug design strategy focused on modification of multiple positions on the polymyxin scaffold and reducing hydrophobicity to optimize antibacterial activity and drug exposure while reducing nephrotoxicity and acute toxicity (9). These innovations make BRII-693 distinctive from polymyxin B and colistin resulting in a novel compound in this class of macrocyclic peptide antibiotics. This paper reports results from a phase 1 study that assessed the safety, tolerability, and PK of BRII-693 in healthy adult participants.

## MATERIALS AND METHODS

### Study design

This was a double-blind, randomized, placebo-controlled, sequential, single ascending dose (SAD), and multiple ascending dose (MAD) study designed to evaluate the safety, tolerability, and PK characteristics of BRII-693 in healthy adult participants (ClinicalTrials.gov identifier NCT04808414). A total of 104 participants were enrolled across seven SAD and five MAD cohorts. Dose escalation to subsequent cohorts was based on a review of safety and tolerability data by a safety review committee prior to starting a new study cohort.

During the SAD phase of the study, cohort dosing of 10, 25, 50, 100, 200, 300, and 400 mg of BRII-693 was evaluated in a sequential manner. Each of the seven SAD cohorts enrolled eight eligible participants (six active; two placebo). On day 1, participants received a single IV dose of BRII-693 or placebo as a 1-hour infusion. Participants were then discharged on day 3.

During the MAD phase of the study, repeat dosing of 100, 150, and 200 mg of BRII-693 was evaluated in sequential cohorts (cohorts 8–10) composed of eight participants each (six active; two placebo). Participants first received a single dose of BRII-693 or placebo as a 1-hour IV infusion on day 1; and then 48 hours later on day 3 through day 9, participants received BRII-693 or placebo every 6 hours (q6h) for 7 days, with the last dose in the morning on day 9. Participants were discharged on day 11. In the remaining two MAD cohorts (cohorts 11 and 12), repeat dosing of 150 mg of BRII-693 q6h for 14 days was evaluated in non-Chinese and Chinese (first and second generation) participants. Each cohort comprises 12 participants (10 active and 2 placebo). The last dose was in the morning on day 14, and participants were discharged on day 17.

Eligible study participants were male or female of non-childbearing potential, aged 18–60 years, and body mass index was within the range of 18.5 and 29.9 kg/m^2^. Participants agreed to refrain from smoking in the previous 48 hours and were deemed medically healthy with clinically insignificant screening results by the principal investigator (e.g., laboratory profiles, medical histories, electrocardiograms [ECGs], and physical examination). Participants were excluded if they had positive testing for HIV, hepatitis B surface antigen, or hepatitis C virus, an excessive history of alcohol intake (more than two drinks for women or four drinks for men per day), received any vaccine or prescription medication (other than hormone replacement therapy for women) within 14 days prior to day 1, use of over-the-counter medication within 7 days prior to day 1, had a creatinine clearance less than 80 mL/min, or had a corrected QT, Fridericia’s formula of greater than 450 for males and greater than 470 for females.

### Safety

Safety assessments included repeated measures of laboratory testing, vital signs, ECGs, physical examinations, and adverse event (AE) monitoring and reporting conducted before, during, and after study drug administration. A >1.5× increase from baseline in creatinine was characterized as meeting the (RIFLE) “Risk” level (or Class R) criteria (10). Participants were confined at the clinical research unit for the duration of their study participation. For the SAD cohorts, confinement was from the day before dosing through day 3 (at least 48 hours after dosing). For the MAD cohorts, confinement was from the day before dosing through day 11 for cohorts 8 to 10 and through day 17 for cohorts 11 and 12.

### Pharmacokinetic assessments

For the SAD cohorts, blood samples for the measurement of BRII-693 in plasma were obtained before dosing and at 0.5 (mid-infusion), 1 (end of infusion), 1.25, 1.5, 2, 4, 6, 8, 12, 24, 36, and 48 hours following the start of dosing infusion. Urine samples were collected for PK assessment before dosing and at the time intervals of 0–4, 4–8, 8–12, 12–24, 24–36, and 36–48 hours after the start of dosing infusion.

For the 7-day MAD cohorts 8–10, blood samples were collected (i) on day 1 before dosing and at 0.5, 1, 1.25, 1.5, 2, 4, 6, 8, 12, 24, 36, and 48 hours following the start of dosing infusion; (ii) on day 3 prior to the morning dose and at 0.5, 1, 1.25, 1.5, 2, 4, and 6 hours after the start of the first dosing infusion; (iii) on day 9 before dosing and at 0.5, 1, 1.25, 1.5, 2, 4, 6, 8, 12, 24, 36, and 48 hours following the start of the last dosing infusion in the morning. Urine samples were collected before dosing and at the time intervals of 0–4, 4–8, 8–12, 12–24, 24–36, and 36–48 hours after the start of dosing infusion on days 1 and 9.

For the 14-day MAD cohorts 11 and 12, blood samples were collected (i) on day 1 prior to and at 0.5, 1, 1.25, 1.5, 2, 4, and 6 hours after the start of the first dosing infusion and (ii) on day 14 before dosing and at 0.5, 1, 1.25, 1.5, 2, 4, 6, 8, 12, 24, 36, 48, 60, and 72 hours following the start of the last dosing infusion in the morning. Urine samples were collected before dosing and at the time intervals of 0–4, 4–8, 8–12, 12–24, 24–36, 36–48, and 48–72 hours after the start of dosing infusion on Day 14.

All blood samples were collected into EDTA-containing vials, immediately placed on ice, and centrifuged at 3,000× g for approximately 10 minutes at 4°C. Supernatant plasma was transferred to two pre-chilled cryopreservation vials (at least 1.0 mL per vial) and stored at −80°C until shipped to the analytical laboratory. Urine samples collected for each time interval were refrigerated, aliquoted, and stored at −80°C until shipped to the analytical laboratory. Plasma and urine samples were analyzed for BRII-693 concentrations using validated high-performance liquid chromatography with detection by mass spectrometry method (BioAgilytix, San Diego, CA). The assay range was 50.0–50,000 ng/mL in both plasma and urine. In plasma, assay precision was 2.95%–5.14%, accuracy was ±1.5% to ±2%, and reproducibility was 97.7%–100%. In urine, assay precision was 1.01%–4.67%, accuracy was ±0.75%, and reproducibility was 98.3%–100%.

### Pharmacokinetic data analyses

Area under the concentration-time curve (AUC) from time zero to the time of the last quantifiable concentration (AUC_0–last_), AUC from time zero to infinity (AUC_0–inf_), AUC within a dosing interval of 6 hours (AUC_0–6h_), maximum observed concentration (*C*_max_), time to reach maximum concentration (*T*_max_), apparent terminal half-life (*t*_1/2_), total clearance (CL), volume of distribution during terminal phase (Vz), and accumulation ratio based on AUC (AR_AUC_) were determined for BRII-693 in plasma. Urine parameters included amount and percentage of dose recovered in urine as unchanged drug over each collection interval and cumulatively and renal clearance (CL_R_). PK parameters were determined using noncompartmental methods in Phoenix WinNonlin (Certara, Version 8.3).

Plasma and urine PK parameters were summarized for each cohort using descriptive statistics. Analyses using linear models were performed to assess dose proportionality (both single-dose cohorts 1–7 and multiple-dose cohorts 8–10). Dose-linearity of C_max_ and AUC across the dose range was assessed by fitting the power model with ln-transformed parameter as response variables and ln-transformed dose (continuous variable) as a fixed effect. For each PK parameter and study phase (SAD or MAD) separately, a pooled estimate (across all dose cohorts) of slope, corresponding 95% confidence interval (CI), and between-subject coefficient of variation (CV) were calculated. Dose proportionality was to be concluded if the 95% CI for the slope spans 1. In addition, to evaluate the effect of ethnicity, C_max_ and AUC from 14-day MAD cohorts 11 (non-Chinese) and 12 (Chinese) were analyzed using a linear fixed effects model, with ln-transformed parameter as response variables and cohort as a fixed effect. Least square means (LSMs) and difference in LSMs with 90% CI were calculated; these values were then back exponentiated to give the geometric LSM, geometric mean ratio, and 90% CI.

## RESULTS

### Demographics

For both the SAD and MAD phases of the study, demographic characteristics were comparable between cohorts. Participant demographics are shown in Tables S1 and S2, respectively. The mean age of participants in the SAD phase was 42.5 years and in the MAD phase was 41.2 years. A majority of the participants were male (SAD: 70.4% and MAD: 74.5%) and white (SAD: 64.8% and MAD: 42.6%). Cohort 12 enrolled only Chinese participants which impacted the racial distribution of the MAD cohorts when compared to the SAD cohorts.

### Safety

#### SAD phase

There were no participants who experienced a serious AE or an AE leading to death during the study. During the SAD phase of the study, 2 of 41 (4.9%) BRII-693-treated participants (1 of 6 [16.7%] participants on BRII-693 100 mg and 1 of 6 [16.7%] participants on BRII-693 200 mg) reported a total of three AEs compared to 1 of 13 (7.7%) placebo participants who reported a total of two AEs. The three AEs in the two BRII-693-treated participants included diarrhea, vomiting, and administration site extravasation which were reported in no more than one participant each. All AEs (five events) in the SAD phase were mild in severity.

#### Seven-day dosing MAD phase

Cohorts 8–10: After single dosing on day 1, a total of 3 of 17 (17.6%) BRII-693-treated participants (1 of 5 [20.0%] participants on BRII-693 150 mg and 2 of 6 [33.3%] participants on BRII-693 200 mg) reported a total of four AEs compared to no AEs reported from placebo participants. The AEs in the BRII-693-treated participants included constipation, vomiting, and headache which were reported in no more than one participant each. Two of the four events were mild in severity (vomiting and constipation), and two were moderate in severity (constipation and headache). One of 6 (16.7%) participants on BRII-693 200 mg discontinued the study due to treatment-related AE of headache of moderate severity after the single dose on day 1.

After multiple dosing, 5 of 17 (29.4%) BRII-693-treated participants (2 of 6 [33.3%] participants on BRII-693 100 mg and 3 of 6 [50.0%] participants on BRII-693 200 mg) reported a total of seven AEs and 3 of 6 (50.0%) placebo participants reported a total of seven AEs. The reported AEs included constipation, abdominal pain, nausea, vomiting, administration site reaction, headache, epistaxis, allergic pruritus, upper abdominal pain, feeling abnormal, and nasal congestion. Constipation was the only AE that was reported in more than one participant in the BRII-693-treated participants. All AEs were mild in severity.

As nephrotoxicity is a class-effect associated with polymyxin B and colistin, creatinine and BUN were measured, and eGFR was calculated to assess renal function in all cohorts. Two of six participants in the highest BRII-693 dose cohort (200 mg q6h × 7 days) had increased serum creatinine above the ULN and corresponding decreases in eGFR. Peak creatinine levels were observed on day 10 (1.54 mg/dL) for one participant and day 9 (1.64 mg/dL) for the other participant. These two participants had a >1.5× increase from baseline in creatinine levels, meeting the RIFLE risk class criteria (or Class R). The creatinine levels in these two participants returned to normal range after completion of therapy on day 11 (1.17 and 1.10 mg/dL, respectively). Dosing was not interrupted in either participant.

#### Fourteen-day dosing MAD phase

Cohorts 11–12: In cohort 11, a total of 5 of 10 (50.0%) BRII-693-treated participants reported a total of 11 AEs compared to 2 of 2 (100.0%) placebo participants, who reported a total of seven AEs. All events were mild in severity. The AEs that were reported in more than one participant in the BRII-693-treated participants included decreased appetite, headache, oral hypoesthesia, and paresthesia.

In cohort 12 (first- or second-generation Chinese participants), 4 of 10 BRII-693-treated participants (40.0%) discontinued the study due to AEs of COVID-19 (two participants), upper respiratory tract infection (one participant), and presyncope (one participant). Five of the 10 (50.0%) BRII-693-treated participants reported a total of eight AEs compared to none of the placebo participants. All events were mild in severity. The AEs reported in more than one participant in the BRII-693-treated participants included administration site reaction and COVID-19. One participant on BRII-693 treatment had an AE of administration site reaction considered treatment-related. One participant had an increase in serum creatinine which met the RIFLE “risk” criteria of >1.5× increase from baseline at days 9 (1.74 mg/dL), 11 (1.72 mg/dL), and 13 (2.07 mg/dL). This participant discontinued the study due to an AE of COVID-19 and could not complete the scheduled day 17 last study visit. At an unscheduled visit after day 13 (last visit completed by the participant), the creatinine level (1.39 mg/dL) was above the ULN and did not meet the RIFLE risk class criteria.

For both the SAD and MAD phases of the study, other than the above serum creatinine data, no clinically significant changes were reported related to laboratory tests, vital signs, ECGs, or physical examination findings.

### Pharmacokinetics

#### SAD phase

**Fig 1:**
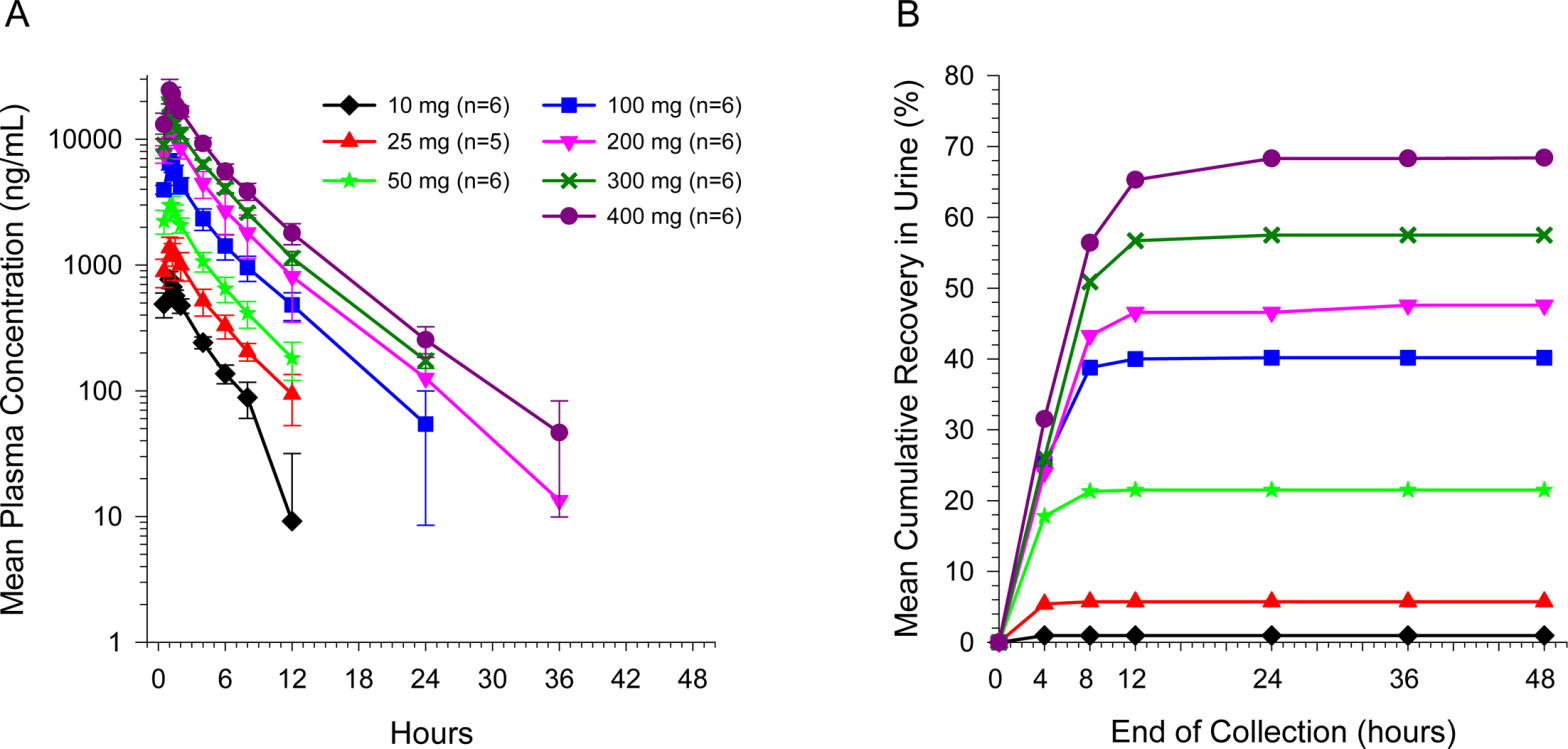
Mean (±SD) plasma concentrations (A) and cumulative urinary recovery (B) of BRII-693 after SADs.

Plasma BRII-693 concentrations increased with increasing dose following single doses of 10–400 mg of BRII-693 by 1 hour IV infusion in healthy adult participants (Fig. 1A). C_max_ was achieved at the end of infusion, and thereafter plasma concentrations declined in a multi-phasic manner. Mean terminal elimination *t*_1/2_ ranged from 2.58 to 4.37 hours and were generally similar across dose levels. Mean total clearance was approximately 4 L/h and appeared to be dose independent (Table 1). *C*_max_, AUC_0–last_, and AUC_0–inf_ increased in a dose-proportional manner with respective slope estimates from regression analysis of 0.953 (95% CI: 0.909, 0.998), 1.02 (95% CI: 0.98, 1.07), and 0.994 (95% CI: 0.952, 1.04), demonstrating that BRII-693 has dose-linear PK characteristics. Between-participant variability was low with geometric CV for C_max_ and AUCs ranging from 14% to 26% and 11% to 27%, respectively.

**TABLE 1:** Arithmetic mean (±SD) PK parameters for BRII-693 after SADs

Parameter	10 mg (*n* = 6)	25 mg (*n* = 5)	50 mg (*n* = 6)	100 mg (*n* = 6)	200 mg (*n* = 6)	300 mg (*n* = 6)	400 mg (*n* = 6)
*C*_max_ (µg/mL)	0.77 ± 0.12	1.50 ± 0.38	3.23 ± 0.46	6.71 ± 0.97	13.2 ± 2.19	17.3 ± 3.05	24.3 ± 4.71
*T*_max_ (h)^*a*^	1.12 (1.08, 1.13)	1.10 (0.72, 1.12)	1.07 (0.50, 1.25)	1.13 (1.13, 1.25)	1.18 (1.10, 1.25)	1.16 (1.07, 1.25)	1.19 (1.07, 1.53)
AUC_0–6h_ (h·µg/mL)	2.08 ± 0.19	4.39 ± 1.13	9.16 ± 1.17	19.2 ± 2.74	36.7 ± 6.29	48.9 ± 6.18	71.4 ± 7.20
AUC_0–inf_ (h·µg/mL)	2.64 ± 0.33	5.94 ± 1.18	12.2 ± 1.79	27.3 ± 4.58	51.4 ± 13.9	69.8 ± 7.75	103 ± 11.6
*t*_1/2_ (h)	2.58 ± 0.46	3.22 ± 0.77	3.20 ± 0.43	4.07 ± 0.61	3.83 ± 1.23	4.12 ± 0.23	4.37 ± 0.51
CL (L/h)	3.83 ± 0.50	4.36 ± 0.94	4.17 ± 0.58	3.76 ± 0.64	4.11 ± 1.04	4.34 ± 0.48	3.93 ± 0.43
V_Z_ (L)	14.1 ± 1.54	20.7 ± 7.61	19.2 ± 3.34	21.8 ± 3.17	21.3 ± 3.27	25.8 ± 2.73	24.6 ± 2.47
fe_0–48h_ (%)	0.95 ± 0.50^*b*^	5.73 ± 3.67	21.5 ± 9.19	40.2 ± 5.40	47.6 ± 15.6	57.5 ± 16.2	68.4 ± 11.4
CL_R_ (L/h)	0.036 ± 0.018^*b*^	0.22 ± 0.12	0.86 ± 0.33	1.51 ± 0.31	1.92 ± 0.80	2.44 ± 0.50	2.68 ± 0.42

^*a*^ Median (range).

^*b*^ *n* = 5.

The mean recovery of BRII-693 in urine over 48 hours as unchanged BRII-693 (fe_0–48h_) ranged from approximately 1%–68% after single doses of 10–400 mg, which increased with dose, especially at lower doses (i.e., up to 100 mg; Table 1). Generally, BRII-693 was mostly excreted by 8 hours following the start of infusion (Fig. 1B). Renal clearance increased in a dose-dependent manner, which was more pronounced at doses up to 100 mg.

#### MAD phase

**Fig 2:**
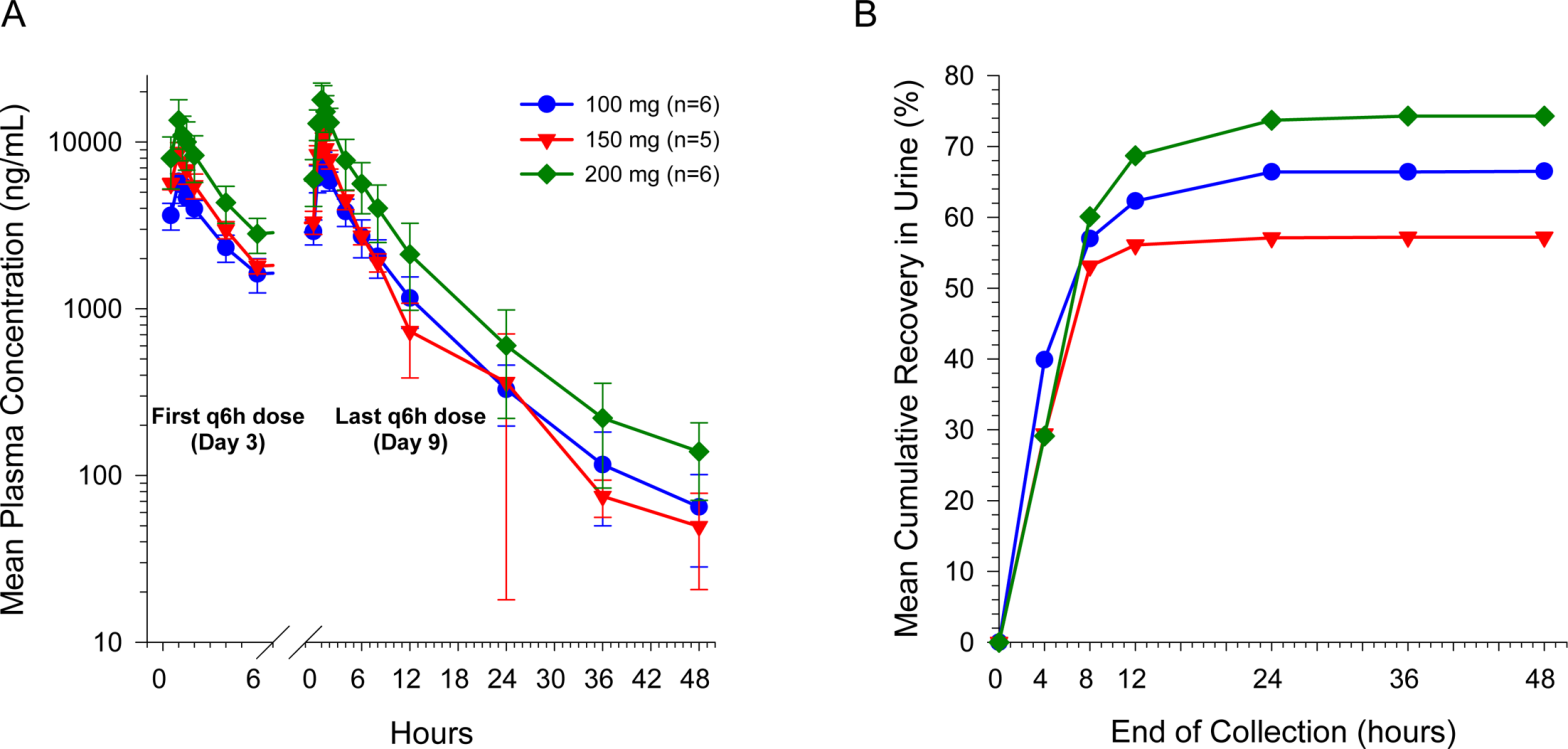
Mean (±SD) plasma concentrations (A) and cumulative urinary recovery (B) of BRII-693 after MADs for 7 days.

On day 1 after single doses of 100, 150, and 200 mg of BRII-693 to healthy adult participants, the PK properties of BRII-693 were very similar to those in the SAD phase. On days 3 through 9, following q6h dosing for 7 days, plasma BRII-693 concentrations increased with increasing dose (Fig. 2A). C_max_ was achieved at end of infusion; mean *t*_1/2_ at steady state (day 9) ranged from 6.76 to 7.58 hours (Table 2), slightly longer than single-dose administration. Mean CL was similar to single-dose administration (approximately 3–4 L/h), suggesting that the PK of BRII-693 is time-independent. Upon q6h dosing for 7 days, BRII-693 accumulated in plasma with mean accumulation ratios of 1.48 to 1.66. Consistent with the observations in the SAD phase, BRII-693 exhibited dose-proportional PK upon multiple dosing; the slope estimates from regression analysis for steady-state (day 9) *C*_max_ and AUC_0–6h_ was 1.13 (95% CI: 0.824, 1.44) and 1.03 (95% CI: 0.644, 1.41), respectively. Between-participant variability remained low after multiple doses.

**TABLE 2:** Arithmetic mean (±SD) PK parameters for BRII-693 after MADs

Parameter	7-day MAD (q6h × 7)			14-day MAD (q6h × 14)	
	100 mg (*n* = 6)	150 mg (*n* = 5)	200 mg (*n* = 5)	150 mg (*n* = 10) (non-Chinese)	150 mg (*n* = 10)^*d*^ (Chinese)
First q6h dose					
*C*_max_ (µg/mL)	5.96 ± 0.71	8.69 ± 1.24	13.5 ± 4.43	9.20 ± 1.05	10.2 ± 1.89
*T*_max_ (h)^*a*^	1.13 (1.08, 1.25)	1.07 (1.02, 1.15)	1.08 (1.05, 1.08)	1.07 (1.03, 1.25)	1.08 (1.02, 1.12)
AUC_0-6h_ (h·µg/mL)	18.0 ± 2.38	24.8 ± 3.45	36.8 ± 10.1	25.2 ± 2.86	27.3 ± 4.14
*t*_1/2_ (h)	2.88 ± 0.50	2.40 ± 0.21	2.49 ± 0.51	2.42 ± 0.29	2.15 ± 0.28
CL (L/h)	4.83 ± 0.43^*b*^	4.94 ± 0.55	4.58 ± 1.14^*c*^	4.80 ± 0.65	4.66 ± 0.73
V_Z_ (L)	17.1 ± 2.72^*b*^	17.2 ± 3.33	15.7 ± 5.81^*c*^	16.7 ± 2.57	14.4 ± 2.93
Last q6h dose					
*C*_max_ (µg/mL)	8.12 ± 1.00	11.7 ± 1.54	18.3 ± 4.15	12.7 ± 1.78	13.3 ± 1.50
*T*_max_ (h)^*a*^	1.18 (1.10, 1.25)	1.07 (1.05, 1.18)	1.10 (1.10, 1.25)	1.07 (1.02, 1.10)	1.07 (1.05, 1.22)
AUC_0–6h_ (h·µg/mL)	28.6 ± 4.48	36.6 ± 4.36	61.3 ± 15.7	39.9 ± 7.15	42.5 ± 4.96
*t*_1/2_ (h)	7.58 ± 1.72	6.76 ± 0.35	7.58 ± 0.93	15.6 ± 11.7	12.4 ± 6.92
CL (L/h)	3.57 ± 0.56	4.15 ± 0.52	3.47 ± 0.99	3.88 ± 0.76	3.57 ± 0.42
V_Z_ (L)	38.4 ± 8.18	40.9 ± 9.47	37.3 ± 8.40	81.4 ± 55.6	61.9 ± 30.2
AR_AUC_	1.59 ± 0.14	1.48 ± 0.10	1.69 ± 0.34	1.58 ± 0.17	1.58 ± 0.19
fe_0–48h_ (%)	66.5 ± 18.0	57.2 ± 19.2	74.3 ± 30.3	74.1 ± 25.6	70.9 ± 22.0
CL_R_ (L/h)	1.40 ± 0.60	1.60 ± 0.60	1.41 ± 0.23	1.91 ± 0.79	1.67 ± 0.43

^*a*^ Median (range).

^*b*^ *n* = 3.

^*c*^ *n* = 4.

^*d*^ *n* = 6 for last q6h dose.

**Fig 3:**
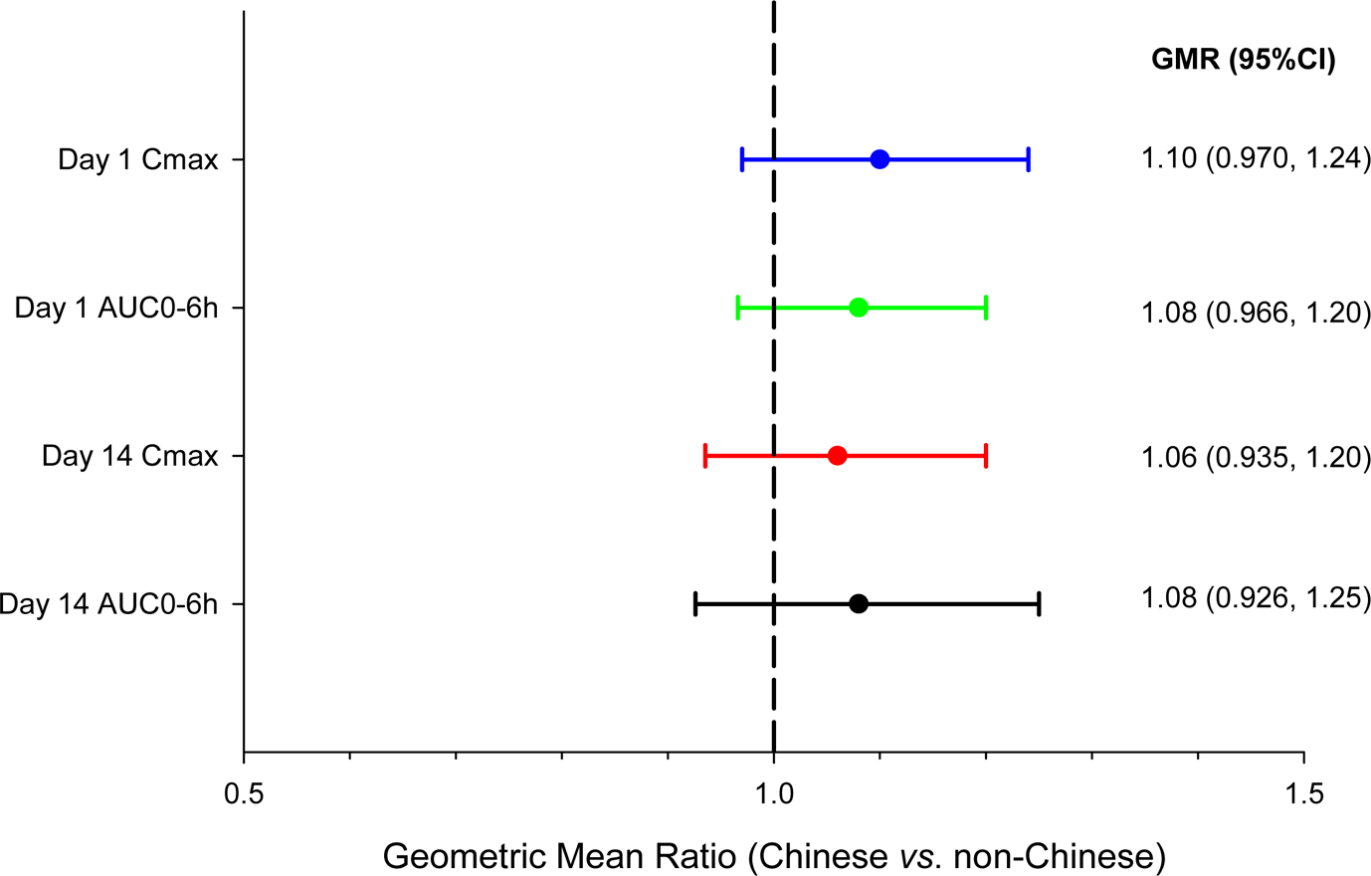
Forest plot for comparison of PK exposures between Chinese and non-Chinese healthy adult participants.

After completion of the 7-day MAD cohorts, two cohorts at 150 mg q6h for 14 days dosing were added with the objectives of confirming the safety and PK exposure at the selected dose and evaluating the ethnicity effect between non-Chinese and Chinese participants. Results showed that the PK profiles of BRII-693 were very similar to those in the 7-day MAD cohorts and between non-Chinese and Chinese participants, apart from an observed longer mean steady state *t*_1/2_ in the 14-day MAD cohorts compared to the 7-day MAD cohorts (12.4–15.6 hours vs 6.76–7.58 hours, respectively), which was due to a few participants who had sustained low concentrations (near assay limit) resulting in a longer terminal phase. Mean CL values were similar across all MAD cohorts (Table 2). Non-Chinese and Chinese participants had statistically comparable PK exposures to BRII-693 (Fig. 3); geometric mean ratios for C_max_ and AUC_0-6h_ were 1.10 (90% CI: 0.970, 1.24) and 1.08 (90% CI: 0.966, 1.20), respectively, after the first dose on day 1, and were 1.06 (0.935, 1.20) and 1.08 (0.926, 1.25), respectively, after the last dose on Day 14. The 90% CIs were well within 0.8–1.25 equivalence limit.

The mean urinary recovery of BRII-693 over 48 hours (fe_0-48h_), after multiple doses (days 9 or 14), was approximately 57%–74%, slightly higher than single doses at the same dose levels (66.5% vs 40.2% for 100 mg, 74.3% vs 47.6% for 200 mg; Tables 1 and 2). The majority of BRII-693 excreted in urine was observed by 8 hours after the start of dosing infusion (Fig. 2B). Non-Chinese and Chinese participants had generally similar urinary recovery profiles for BRII-693.

## DISCUSSION

Safety, tolerability, and pharmacokinetics have been evaluated at single and multiple doses of BRII-693 in healthy adult participants. Results from this study have demonstrated that BRII-693 was safe and well tolerated following IV infusion over 1 hour at single doses ranging from 10 to 400 mg and multiple doses of 100, 150, and 200 mg every 6 hours for 7–14 days. None of the reported AEs were severe or serious, there was no evidence of increasing severity of AEs with increasing dose, and the majority of AEs were of mild severity across all cohorts and for both non-Chinese and Chinese participants.

Reversible creatinine elevations were observed in some participants in the higher dosing cohorts with BRII-693: 2 of 6 participants receiving BRII-693 200 mg q6h × 7 days and 1 of 20 participants receiving BRII-693 150 mg q6h × 14 days. There was no oliguria or other acute kidney injury-related AEs noted during the conduct of the study. There were also no other safety concerns regarding laboratory tests, vital signs, ECGs, or physical examinations. As this was a phase 1 study with a limited number of healthy participants, further studies are warranted to further evaluate the safety of BRII-693.

PK results showed that BRII-693 was rapidly eliminated after the end of infusion, with mean *t*_1/2_ of approximately 3–4 hours after single dose and 7–8 hours after multiple doses. BRII-693 exhibited dose-proportional PK profile over the range of doses studied in both SAD and MAD phases and time-independent PK characteristics. With the approximately 3–8 hours mean *t*_1/2_, a dosing interval of every 6 hours, and the observed time-independence in PK, BRII-693 was expected to accumulate in plasma with an accumulation ratio of 1.3 to 2.5 at steady state, which generally aligns with the observed value of 1.5–1.7. The PK exposure to BRII-693 was statistically equivalent between non-Chinese and Chinese participants. Analysis of urine PK samples showed that BRII-693 had higher urinary recovery than polymyxin B (11), and urinary excretion was dose dependent across single- and multiple-ascending-dose cohorts. At the same dose level (i.e., 100 and 200 mg cohorts), urinary excretion of BRII-693 in the MAD phase at steady state was higher than that in the SAD phase after a single dose, and approximately 60%–70% of the dose was excreted as BRII-693.

BRII-693 emerged from an extensive discovery program where optimization of multiple positions throughout the polymyxin scaffold was targeted demonstrating increases *in vitro* and *in vivo* potency and a potentially improved safety profile compared to polmyxin B (9). These phase 1 clinical trial data, presented here, will be useful for determining dose and regimen for future clinical studies of BRII-693.

## SUPPLEMENTAL MATERIAL

Supplemental material aac.01288-24-s0001.docx Tables S1 and S2; Figures S1 to S4.

ASM does not own the copyrights to Supplemental Material that may be linked to, or accessed through, an article. The authors have granted ASM a non-exclusive, world-wide license to publish the Supplemental Material files. Please contact the corresponding author directly for reuse.
