# Supplementary material for: Phase 1 study of the safety, tolerability, and pharmacokinetics of a synthetic macrocyclic peptide antibiotic (BRII-693) in healthy adult participants
Source: Antimicrob Agents Chemother. 2024 Dec 9;69(1):e01288-24. doi: 10.1128/aac.01288-24 (PMC11784026; doi:10.1128/aac.01288-24)
Supplement: Supplemental material — Tables S1 and S2; Figures S1 to S4. [file aac.01288-24-s0001.docx]

**SUPPLEMENTAL MATERIALS**

Phase 1 Study of the Safety, Tolerability, and Pharmacokinetics of Next Generation Polymyxin

(BRII-693) in Healthy Adult Participants

## Michael Watkins,1# Yali Zhu,1 David C. Griffith,2 Jeffery S. Loutit,2 David Margolis,1 Peidi Gu1

1 Brii Biosciences Inc, Durham, NC, United States

2 Qpex Biopharma, Inc., San Diego, California, United States

**TABLE S1**. Demographics by Treatment Cohort (SAD Phase)

| **Parameter** | **Pooled**  **placebo** | **Cohort 1**  **BRII-693**  **10 mg** | **Cohort 2**  **BRII-693**  **25 mg** | **Cohort 3**  **BRII-693**  **50 mg** | **Cohort 4**  **BRII-693**  **100 mg** | **Cohort 5**  **BRII-693**  **200 mg** | **Cohort 6**  **BRII-693**  **300 mg** | **Cohort 7**  **BRII-693**  **400 mg** | **Total** |
| --- | --- | --- | --- | --- | --- | --- | --- | --- | --- |
| No. participants | 13 | 6 | 5 | 6 | 6 | 6 | 6 | 6 | 54 |
| Age (years) |  |  |  |  |  |  |  |  |  |
| Mean | 39.4 | 41.2 | 43.0 | 42.3 | 49.3 | 41.0 | 42.5 | 45.3 | 42.5 |
| SD | 12.11 | 13.17 | 17.46 | 12.83 | 9.31 | 15.99 | 13.95 | 8.52 | 12.44 |
| Min, Max | 22, 59 | 22, 57 | 19, 58 | 24, 57 | 39, 60 | 21, 60 | 27, 60 | 34, 57 | 19, 60 |
| Sex |  |  |  |  |  |  |  |  |  |
| Male | 10 (76.9) | 3 (50.0) | 4 (80.0) | 4 (66.7) | 3 (50.0) | 3 (50.0) | 5 (83.3) | 6 (100) | 38 (70.4) |
| Female | 3 (23.1) | 3 (50.0) | 1 (20.0) | 2 (33.3) | 3 (50.0) | 3 (50.0) | 1 (16.7) | 0 | 16 (29.6) |
| Race |  |  |  |  |  |  |  |  |  |
| Asian | 2 (15.4) | 1 (16.7) | 1 (20.0) | 0 | 0 | 1 (16.7) | 0 | 0 | 5 (9.3) |
| Black or African American | 2 (15.4) | 0 | 1 (20.0) | 2 (33.3) | 3 (50.0) | 1 (16.7) | 2 (33.3) | 1 (16.7) | 12 (22.2) |
| White | 9 (69.2) | 5 (83.3) | 3 (60.0) | 3 (50.0) | 3 (50.0) | 3 (50.0) | 4 (66.7) | 5 (83.3) | 35 (64.8) |
| Multiple | 0 | 0 | 0 | 0 | 0 | 1 (16.7) | 0 | 0 | 1 (1.9) |
| Other | 0 | 0 | 0 | 1 (16.7) | 0 | 0 | 0 | 0 | 1 (1.9) |
| BMI (kg/m^2^) |  |  |  |  |  |  |  |  |  |
| Mean | 26.5 | 24.9 | 26.0 | 26.8 | 26.6 | 27.4 | 25.4 | 26.2 | 26.3 |
| SD | 2.95 | 2.23 | 2.47 | 1.78 | 2.54 | 1.90 | 3.61 | 1.88 | 2.50 |
| Min, Max | 22.2, 29.8 | 21.5, 27.8 | 21.9, 28.4 | 24.7, 28.4 | 22.1, 29.3 | 24.9, 29.8 | 19.5, 28.7 | 22.9, 28.2 | 19.5, 29.8 |

BMI = Body mass index; Max = Maximum; Min = Minimum; MAD = Multiple ascending dose; SD = Standard deviation.

**TABLE S2**. Demographics by Treatment Cohort (MAD Phase)

| **Parameter** | **Pooled**  **placebo** | **Cohort 8**  **BRII-693**  **100 mg** | **Cohort 9:**  **BRII-693**  **150 mg** | **Cohort 10:**  **BRII-693**  **200 mg** | **Cohort 11**  **BRII-693**  **150 mg** | **Cohort 12**  **(Chinese)**  **BRII-693**  **150 mg** | **Total** |
| --- | --- | --- | --- | --- | --- | --- | --- |
| No. participants | 10 | 6 | 5 | 6 | 10 | 10 | 47 |
| Age (years) |  |  |  |  |  |  |  |
| Mean | 36.1 | 55.0 | 38.0 | 45.5 | 41.7 | 36.7 | 41.2 |
| SD | 12.64 | 5.14 | 13.82 | 13.13 | 9.30 | 9.72 | 11.97 |
| Min, Max | 19, 56 | 46, 60 | 23, 59 | 26, 59 | 30, 60 | 25, 56 | 19, 60 |
| Sex |  |  |  |  |  |  |  |
| Male | 6 (60.0) | 5 (83.3) | 4 (80.0) | 4 (66.7) | 7 (70.0) | 9 (90.0) | 35 (74.5) |
| Female | 4 (40.0) | 1 (16.7) | 1 (20.0) | 2 (33.3) | 3 (30.0) | 1 (10.0) | 12 (25.5) |
| Race |  |  |  |  |  |  |  |
| Asian | 3 (30.0) | 0 | 0 | 1 (16.7) | 0 | 10 (100) | 14 (29.8) |
| First Generation Chinese | 2 (20.0) | 0 | 0 | 0 | 0 | 9 (90.0) | 11 (23.4) |
| Second Generation Chinese | 0 | 0 | 0 | 0 | 0 | 1 (10.0) | 1 (2.1) |
| Black or African American | 3 (30.0) | 1 (16.7) | 1 (20.0) | 2 (33.3) | 3 (30.0) | 0 | 10 (21.3) |
| White | 4 (40.0) | 5 (83.3) | 3 (60.0) | 2 (33.3) | 6 (60.0) | 0 | 20 (42.6) |
| Multiple | 0 | 0 | 1 (20.0) | 1 (16.7) | 1 (10.0) | 0 | 3 (6.4) |
| BMI (kg/m^2^) |  |  |  |  |  |  |  |
| Mean | 26.2 | 27.7 | 28.7 | 26.1 | 27.2 | 25.1 | 26.6 |
| SD | 2.46 | 1.77 | 0.95 | 2.27 | 1.10 | 2.57 | 2.23 |
| Min, Max | 21.3, 29.6 | 25.2, 29.6 | 27.2, 29.7 | 22.3, 29.1 | 25.6, 29.2 | 19.3, 29.3 | 19.3, 29.7 |

BMI = Body mass index; Max = Maximum; Min = Minimum; MAD = Multiple ascending dose; SD = Standard deviation.

**Figure S1-1: Mean (+SD) Serum Creatinine Over Time**

**Figure S1-2: Individual and Mean Serum Creatinine Over Time**

**Figure S2-1: Mean (+SD) Blood Potassium Levels Over Time**

**Figure S2-2: Individual and Mean Blood Potassium Levels Over Time**

**Figure S3-1: Mean (+SD) Blood Sodium Levels Over Time**

**Figure S3-2: Individual and Mean Blood Sodium Levels Over Time**

**Figure S4-1: Mean (+SD) Blood Chloride Levels Over Time**

**Figure S4-2: Individual and Mean Blood Chloride Levels Over Time**
